# Retinoic Acid: A New Old Friend of IL-17A in the Immune Pathogeny of Liver Fibrosis

**DOI:** 10.3389/fimmu.2021.691073

**Published:** 2021-06-15

**Authors:** Daria M. Kartasheva-Ebertz, Stanislas Pol, Sylvie Lagaye

**Affiliations:** ^1^ Institut Pasteur, INSERM U1223, Paris, France; ^2^ Université de Paris, Paris, France; ^3^ APHP, Groupe Hospitalier Cochin, Département d’Hépatologie, Paris, France

**Keywords:** IL-17A, retinoic acid, human liver fibrosis, chronic liver inflammation, fibrosis resolution

## Abstract

Despite all the medical advances mortality due to cirrhosis and hepatocellular carcinoma, the end stages of fibrosis, continuously increases. Recent data suggest that liver fibrosis is guided by type 3 inflammation with IL-17A at the top of the line. The storage of vitamin A and its active metabolites, as well as genetics, can influence the development and progression of liver fibrosis and inflammation. Retinoic acid (active metabolite of vitamin A) is able to regulate the differentiation of IL-17A^+^/IL-22–producing cells as well as the expression of profibrotic markers. IL-17A and its pro-fibrotic role in the liver is the most studied, while the interaction and communication between IL-17A, IL-22, and vitamin A–active metabolites has not been investigated. We aim to update what is known about IL-17A, IL-22, and retinoic acid in the pathobiology of liver diseases.

## Highlights

- Type 3 inflammation (Th17, Tc17 cells) is involved in the process that guides liver fibrosis.
- Hepatic stellate cells activation is directly linked to the release of vitamin A from lipid droplets that are used as an important energy source for myofibroblast transformation, supported by autophagy.
- There are the SNP variants coding vitamin A metabolizing enzymes (PNPLA3, HSD17B13), associated with opposite prognosis in several chronic liver diseases.
- Immune modulation of the IL-17A/RA axis could be a new important component of the very active therapeutic developments of NASH and fibrotic disease.

## Introduction

Liver fibrosis is the obligatory result of chronic liver disease, from which approximately 2 million people die each year (1). Despite all the medical advances, mortality due to cirrhosis and hepatocellular carcinoma (HCC), the end stages of fibrosis, continuously increases (2). The background of liver fibrosis involves multiple processes, including alcoholic lesions, chronic forms of viral hepatitis, and metabolic syndrome, which is becoming one of the most important causes today, a burden on our society. Western diets, reduced physical activity, and constant stress all actively contribute to the dramatic increase in nonalcoholic steato-hepatitis (NASH) prevalence (3, 4).

The liver is known to be the main storage site for vitamin A and its derivatives. Vitamin A is a powerful antioxidant; its active metabolites directly regulate gene expression, participate in differentiation of IL-17A**^+^**/IL-22^+^ cells. Any impairment in retinoic acid (RA) metabolism probably affects the metabolic and immunological pattern of liver disease and, in particular, progression to fibrosis. Recent studies on the genetic substrate of liver diseases, particularly nonalcoholic fatty liver disease (NAFLD), have highlighted the presence of associated SNPs in genes that are involved in vitamin A metabolism. We propose to trace the interaction of three molecules (IL-17A, IL-22, RA) and assess the possible impact of this trio on the development of liver fibrosis. There may be a need to study the interaction of vitamin A metabolites and the immune component of the liver in the development of fibrosis, and this will provide new options for fibrosis treatment.

## Liver Homeostasis and Inflammation

The main causes of liver fibrosis are viral hepatitis, alcoholic, and nonalcoholic fatty liver disease, as well as cholestatic liver disease (1). If viral hepatitis could be controlled by anti-viral therapy and vaccination, alcoholic disease by abstinence from alcohol, the trends of recent years indicate a clear increase of the part of NAFLD in liver diseases and fibrosis, respectively. This is related to lifestyle factors, reduced physical activity, consumption of excessive amounts of fats and sugar, and great difficulty in changing lifestyle and habits, which is required in the treatment of this pathology (4).

In the genesis of liver disease, a huge role belongs to the development of chronic inflammatory response. The liver microenvironment determines the balance between tolerance and inflammation in the healthy organ (5). The blood carries large amounts of intestinal antigens detected by pattern recognition receptors (PRRs) located on liver resident macrophages (6) or hepatocytes (7) that have to be neutralized. After PRR stimulation, antigens are degraded silently, without usual secretion of pro-inflammatory cytokines (5). Such silent blood detoxification protects the body from massive activation of the immune system in response to microbes from the gut. In healthy subjects, such a process does not go beyond homeostasis. However, under the influence of various factors, the immunological hepatic tolerance is broken, followed by inflammation, and the tissue regeneration processes are disturbed. The hepatic stellate cells (HSC) lose lipid droplets of vitamin A and trans-differentiate into myofibroblasts (8), secreting extracellular matrix (ECM). The HSCs are located in the space of Disse, the space between hepatic trabeculae and sinusoids. The space of Disse is separated from the sinusoids by liver sinusoidal endothelial cells (LSECs). HSCs are the main cells responsible for the initiation of fibrosis, producers of extracellular matrix (9). In their inactive state, they express neuronal markers and are the main site of vitamin A storage in lipid droplets in the body. During activation, HSCs lose the expression of neuronal markers, as well as lipid droplets and transdifferentiate into myofibroblasts (8), cells with high proliferative and migratory potential. Myofibroblasts migrate to the site of inflammation and increase the expression of mesenchymal markers, such as α-SMA or type 1 collagen.

The massive and constant ECM production distorts the hepatic and vascular architecture leading to cirrhosis and hepatocarcinoma and may require liver transplantation. Although hepatocarcinoma may occur in the absence of advanced fibrosis stages (10).

In any case, inflammation precedes fibrosis. It appears that the branch of CD4**^+^**T-lymphocytes, the Th17 population (11), seems to be involved in the inflammation process that guides liver fibrosis and underlining liver pathologies (12–15). This branch is composed of Th17 CD3CD4**^+^** or Tc17 CD3CD8**^+^** cells, expressing the RORγτ transcription factor (16), and secreting IL-17A alone or in combination with IL-22, as two signature cytokines of this population.

### Biology of IL-17A

The main source of IL-17A is the Th17 CD4^+^ T lymphocytes (LTs) (11). Other cell populations may be involved: the CD8**^+^** (Tc17) LTs (17–19), double-negative LTs, LTγδ, NKT cells, ILC_3_ cells, MAIT cells, monocytes, and even neutrophils (20). IL-17A expression in liver HSC cells has also been reported (21). IL-17A receptor (IL-17AR) expression is ubiquitous with the highest levels occurring in hematopoietic cells, while the major responses to IL-17A occur in epithelial, endothelial, and fibroblast cells (22). IL-17 receptor family includes five IL-17RA to IL-17RE receptor subunits. IL-17A signaling is mediated predominantly through the IL-17RA and IL-17RC subunits (23). In the liver, in addition to immune cells, IL-17AR expression has been detected on all types of hepatic cells, including hepatocytes, HSCs, biliary epithelial cells, and LSECs (24).

IL-17A activates a highly pro-inflammatory gene expression program, typical of that induced by innate immune receptors, such as IL-1R and TLRs (25), using the Act1 adapter instead of TRIF/Myd88, but similarly activates the nuclear factor κB (NFκB), MAPK, C/EBPβδ pathways (22).

IL-17A is a driver of hematopoietic cell differentiation in the bone marrow to the granulocyte lineage by direct stimulation of granulocyte colony-stimulating factor (GM-CSF) expression by epithelial cells and STAT3 activation (26, 27). At the same time, IL-17A in cooperation with TNF-α stimulates the expression of adhesive molecules on endothelial cells, such as Selectin-E or ICAM-1, making possible enhanced granulocyte migration (28), which is increased by IL-8 secretion, that acts as an attractant for neutrophils (29, 30). A similar mechanism is involved in the initiation and development of liver fibrosis and is a part of carcinogenesis of certain tumors.

IL-17A neutralization is effective in psoriasis, rheumatoid arthritis, ankylosing spondylitis (31, 32), but not Crohn’s disease where it increases inflammation and susceptibility to fungal infections (33). Moreover, in the experimental autoimmune encephalomyelitis (EAE), an autoimmune model, not all Th17 cells have destructive autoimmune properties. Th17 generated under the influence of TGF-β1 and IL-6 produce IL-17A, and it does not induce autoimmune events without receiving additional stimulation by IL-23. However, Th17 generated under the influence of TGF-β3 do not need to receive IL-23 signal to be pathogenic. The molecular signature of these two Th17 populations is different (34). Neutralization of IL-23 improves liver fibrosis in the bile-duct ligation (BDL) mouse model (14). It turns out that the complete acquisition of the pathogenic function of Th17 is mediated by IL-23 rather than by TGF-β1 and IL-6 (35). All this suggests that IL-17A behaves differently depending on its tissue localization and the environment in which it acts.

### Biology of IL-22

IL-22 is a cytokine of the IL-10 family, secreted by a vast majority of hematopoietic cells: Th17, Tγδ, ILC3, NKT lymphocytes (36). Signaling occurs *via* the interferon family receptor IL-22R that binds with a second CRF2-4 component (IL10R2) shared with IL-10 (37, 38). An IL-22–binding protein (IL-22BP), a soluble molecule, binds IL-22 and blocks its interaction with the receptor complex, thereby preventing activation (39).

IL-22R is expressed on stromal and epithelial cells in various organs, including liver (36, 40); however, immune cells do not express it, and IL-22 does not appear to affect them (41). Downstream of the IL-22 receptor complex is the JAK-STAT pathway leading to the STAT1, STAT3, and STAT5 phosphorylation. In addition, IL-22 is capable of activating the three main MAPK pathways: the MEK-ERK-RSK, the JNK/SAPK, and the p38 kinase pathways (42). Unlike the majority of cytokines that target different cell types, the unique target of IL-22 is the non-hematopoietic cells of epithelia. IL-22 is a part of the established inflammation, but regulates tissue processes, a true regulator of epithelia.

### Retinoic Acid

**Figure 1:**
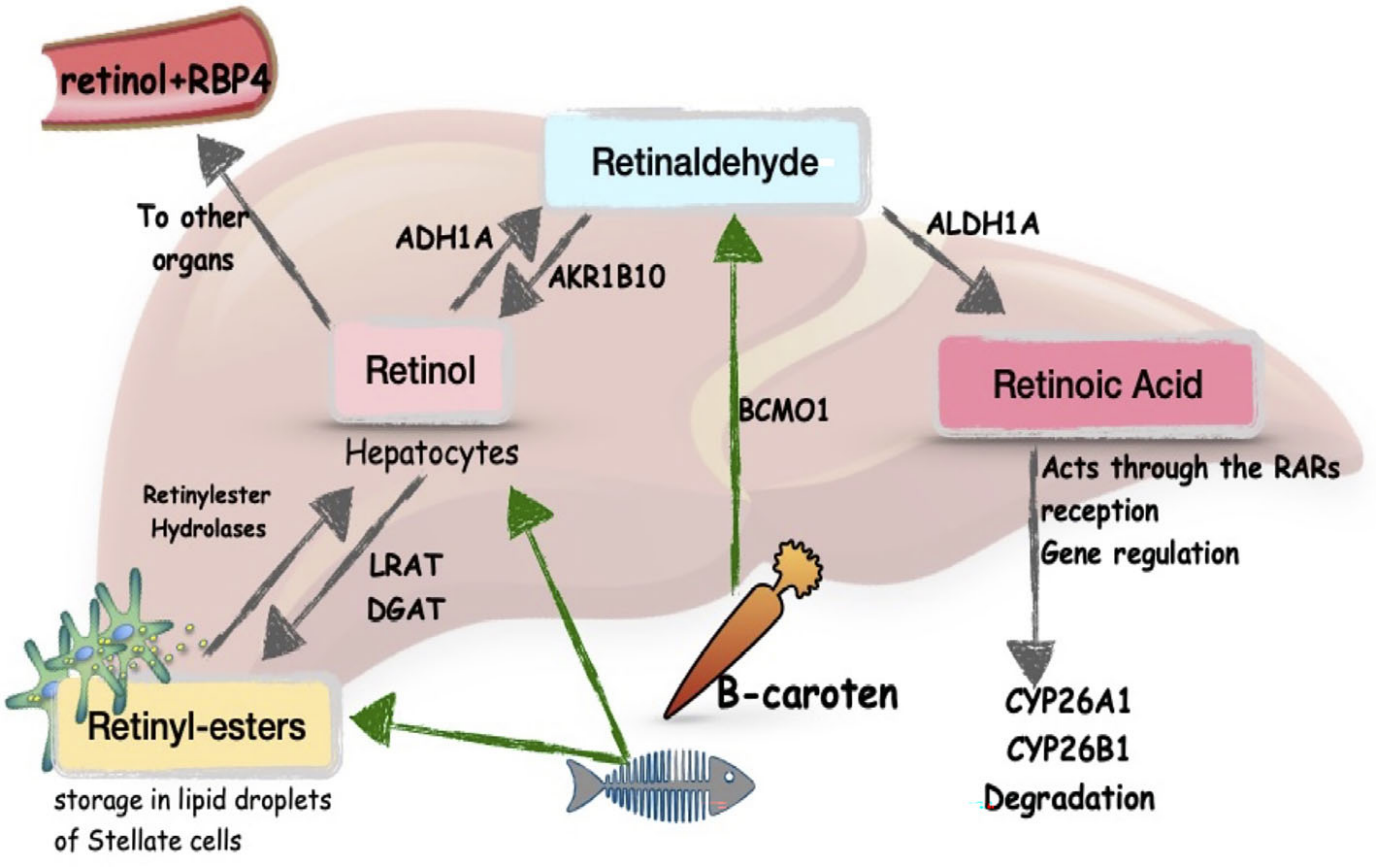
Metabolism of vitamin A in the liver. Schema modified from Blaner, 43 and Pettinelli et al., 44. LRAT, lecithin retinol acyltransferase; DGAT, diglyceride acyltransferase; ADH1A, alcohol dehydrogenase 1A; AKR1B10, aldo-keto reductase family 1 member B10; BCMO1, beta-carotene monooxygenase; ALDH1A, aldehyde dehydrogenase 1 family member A; RBP4, retinol binding protein 4.

RA is an active metabolite of vitamin A which is liposoluble. Vitamin A is stored in its esterified form, retinyl esters, in the HSCs, which mainly trigger liver fibrosis after activation. Retinaldehydrogenases catalyze the release of active metabolites of vitamin A (**Figure 1**), including RA (43, 45). Vitamin A is distributed to tissues in the form of retinol, by retinol binding protein 4 (RBP4), produced in the liver. RA regulates the expression of several hundred genes, and this is what provides most of its functions (45). RA acts through nuclear receptors: RA receptors (RARα, β, γ) and retinoid X receptors (RXRα, β, γ). The receptors form homo- or heterodimers and exert their action by binding with the RA response element (RARE) in the promoters of regulated genes (46).

Despite the storage of vitamin A in the liver, RA plays a critical role in the regulation and maintenance of intestinal epithelium on the one hand (47), and mucosal immunological function on the other (48). NAFLD is directly related to a permeability disorder of the intestinal epithelium. Disruption of vitamin A metabolism and RA signaling will affect both hepatic functionality and intestinal integrity, creating a vicious circle of events (43). It is still unknown exactly what is primary and to what extent disruption of vitamin A metabolism impacts the immunological environment in the liver, the metabolic environment, and would promote the progression of liver fibrosis.

RA is known to influence FoxP3^+^Tregs and Th17 differentiation, to induce intestinal homing of innate lymphoid cells (ILC) (49), to stimulate the secretion of pro-inflammatory cytokines during infections, and in synergy with dendritic cells to sensitize effector lymphocytes (50, 51).

### Th17 Differentiation: The Role of RA

**Figure 2:**
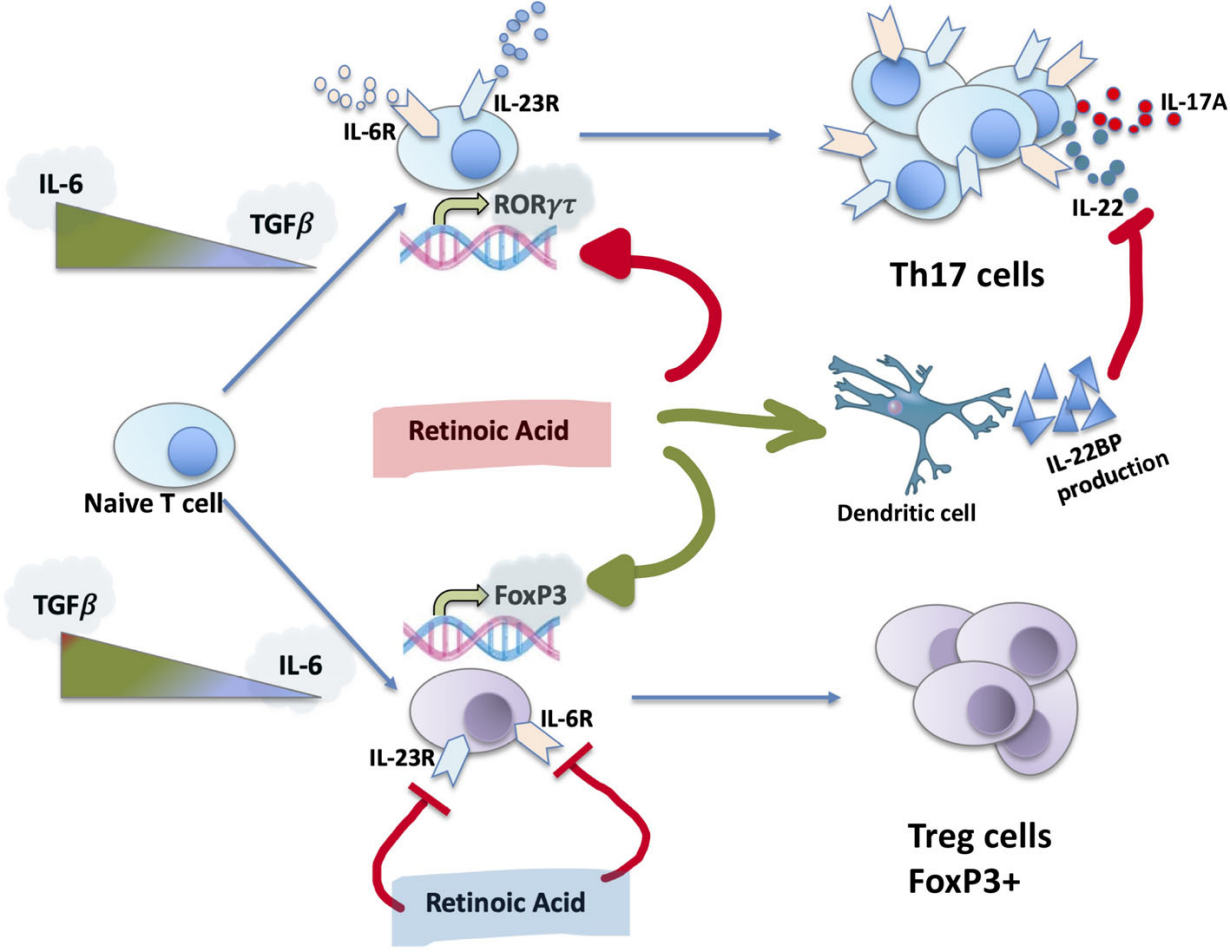
Th17 differentiation, role of RA. RA, the active metabolite of vitamin A, is capable to inhibit RORγτ expression, promoting the development of iTregs and FoxP3 expression. RA inhibits the expression of IL-23 and IL-6 receptors, stimulates IL-22BP synthesis by dendritic cells.

Th17 cells are considered to be the main source of the cytokines IL-17A and IL-22 (52). The differentiation and expansion of Th17 from naive LTs are dependent on two cytokines: TGF-β1 and IL-6 (**Figure 2**). This process is regulated by IL-23 and IL-21 (52). High concentrations of IL-6, secreted by macrophages, in its STAT3-dependent manner will activate HIFα, which in turn will target FoxP3 for ubiquitination and proteasomal degradation (53), promoting the expression of the RORγt factor and the Th17 branch (54). Low concentration of TGF-β1 in cooperation with IL-6 will induce the development of Th17s and the expression of the IL-23R receptor (54), while a high concentration in the absence of IL-6 will promote the iTreg lineage (53). This Treg/Th17 counterbalance is used in research as a marker of type 3 inflammation. IL-23, which belongs to the IL-12 family of cytokines, acts as a stabilizer that is essential for the correct development of Th17 (55). During inflammation IL-23 is produced by activated dendritic cells and, by acting on T lymphocytes, increases IL-17A secretion (56).

In addition to IL-6, TGF-β1, and IL-23, the expression of IL-17A is regulated by retinoids, in particular RA (**Figure 2**), which is important in the context of liver disease and fibrosis, since the liver is the main site of storage of Vitamin A. RA is capable of inhibiting the expression of RORγτ, promoting the development of iTregs and the expression of FoxP3. This occurs in an independent manner of STAT3/STAT5 and IL-2 signaling (57, 58). Schambach et al. provided evidence that the effects of active vitamin A metabolites are likely to be at least partially mediated by the nuclear RARα (59). Moreover, RA induces IL-22BP expression in monocyte-derived dendritic cells. In addition to direct regulation of Th17 cells formation, RA separately regulates the action of IL-22 (60).

In *in vitro* models, RA is very efficient in the generation of iTregs despite Th17 (61). RA enhances TGF-β1-signaling by increasing SMAD-3–dependent FoxP3 expression even in the presence of IL-6 (61). However *in vivo* there is no increase in the frequency of iTregs, whereas RA suppresses the EAE by inhibiting the inflammatory action of Th17 through the inhibition of IL-6Ra, IL-23R, and interferon regulatory factor 4 (IRF-4) receptor expression on effector T cells (61).

### Genetic Associations, Vitamin A, and Chronic Liver Diseases

Analysis of gene expression involved in vitamin A metabolism showed that aldo-keto reductase family 1 member B10 (AKR1B10), the enzyme converting all-trans-retinaldehyde to retinol (**Figure 1**), is up-regulated in NASH patients compared with healthy controls, which is associated with elevated blood retinol levels (44). In contrast, the enzymes known to convert retinaldehyde to RA: aldehyde dehydrogenase 1 family member A (ALDH1A1, ALDH1A2, ALDH1A3), exhibit decreased expression in NASH patients (44)^-^ (62). However, CYP26A1 and CYP26B1 expression is enhanced in NASH patients, indicating more intensive degradation of RA (62). Consequently, this impairs the availability of active vitamin A metabolites in the liver, particularly RA, altering the functioning of signaling pathways.

Borel and Desmarchelier, 2017 have reviewed genetic variations thought to be associated with modulation of vitamin A blood levels (63). These include mutations and SNPs in RBP4, beta-caroten oxygenase 1 (BCO1), scavenger-receptor class B 1 (SCARB1), APOB, CXCL8, CD36, and other genes. For the moment, there are just some variants in genes that encode important enzymes, associated with the progression of chronic liver diseases, particularly the progression of NAFLD to fibrotic or cirrhotic liver and HCC (64) (reviewed by Carlsson, 2020). Some of them are presumably involved in vitamin A metabolism.

PNPLA3 is a member of a family of patatine-domain containing lipid hydrolases, localizes to the surface of lipid droplets, with multiple substrates, including triacylglycerols, phospholipids and retinols-esters, the storage form of vitamin A. PNPLA3 is thought to be involved in release of retinol in response to insulin from HSC (65), an important step of HSC activation. The first SNP found to be associated with NASH progression to advanced fibrosis or HCC is PNPLA3 I148M variant, associated with decreased enzymatic activity, increased hepatic fat storage, progression of steatohepatitis, elevated plasma liver enzymes, fibrosis. At the same time, in PNPLA3 I148M minor allele carriers, the storage of liver retinyl-palmitate is increased, and the ratio of retinol/retinyl-palmitate is reduced (66), which reveals a disturbance in the vitamin A metabolism and signaling. PNPLA3148M SNP carriers with obesity and fatty liver disease or who are just obese, have lower levels of circulating retinol, as well as RBP4 protein concentration (67). Meanwhile, Blaner questions the hydrolase activity of PNPLA3, arguing for insufficient data (43). This needs to be explored.

HSD17B13 is the second protein of interest with restricted expression for hepatocytes, which belongs to the short-chain dehydrogenase/reductase family, involved in the metabolism of steroid hormones, prostaglandins, lipids, and xenobiotics. Biological function of HSD17B13 is not clear: when overexpressed, the size and number of lipid droplets in hepatocytes is increased (68), but this remains debated (69). Meanwhile, hepatic expression of HSD17B13 is higher in NASH patients compared with healthy individuals (70). P260S mutation (rs62305723) in *HSD17B13* gene, abolishing retinol dehydrogenase activity *in vitro*, is associated with decreased inflammation and ballooning (70). Another loss-of-function variant in *HSD17B13* (rs72613567:TA) was found to be associated with a reduced risk of chronic liver disease, like alcoholic and non-alcoholic chronic liver diseases, reduced risk of progression from steatosis to steatohepatitis, but not simple steatosis, as well as reduced risk of alcoholic and non-alcoholic cirrhosis in allele dose-dependent manner (69) ^(^ 71 ^),^. However, an attempt to replicate the protective effect in the whole body knock out (KO) for HSD17B13 murine model failed. No difference was observed between the KO model and the wild type (WT) genotype neither in the severity of liver damage due to a high-fat diet or alcohol consumption model nor in the rate of disease progression to cirrhosis and hepatocarcinoma (72).

## IL-17A, IL-22, and RA in Liver Fibrosis

Fibrosis is a two-way process, capable of regressing even in advanced stages (73). Liver fibrosis of any etiology predisposes to the development of hepatocarcinoma (74), which may also occur on a background of chronic inflammation without advanced fibrosis (75, 76). The exact immunological mechanisms that direct the development or regression of fibrosis, or development of hepatocarcinoma without adjacent fibrosis, are not well elucidated.

### IL-17A, IL-22, and Acute Liver Injury

#### IL-17A

During acute liver injury Kupffer cells, found in the sinusoids and secreting platelet derived growth factor (PDGF), TNFα, IL-6, IL-1β, TGF-β1 (77), stimulate STAT3 signaling in HSCs. HSC awaken from their quiescent state, release their vitamin A stock and transform into myofibroblasts, which migrate to the site of injury (8). In the injury, INFγ, the potent STAT1 activator, secreted by intrahepatic T cells, induces apoptosis of damaged/infected hepatocytes amplifying the inflammatory signal (78, 79). While IL-6–directed response, ensured by STAT3 expression in myeloid cells, protects against liver damage by counteracting INFγ-signaling (80). Meanwhile the promotion of STAT3 expression under TGF-β1 and IL-6 also stimulates the Th17 phenotype. IL-17A can stimulate STAT3 phosphorylation directly (*in vitro*) or *via* enhancing IL-6 secretion (81). In the case of acute viral diseases, IL-17A, on the one hand, can sensitize antigen-specific effector T cells (82), promoting the elimination of the agent, on the other hand, an exhaustive IL-17A and IL-6 response can promote viral persistence (83).

IL-17A neutralization aggravates the development of acute hepatitis in a-galactosylceramide model directed by IL-17A**^+^**NKT cells (84). Similarly, TγδIL-17A^+^-depleted HBs**^+^** transgenic mice during the concanavalin A (ConA) lesion, develop inflammation exacerbated by INFγ and accompanied by potent necrosis. Injection of IL-23 decreased liver damage (85). However, in WT mice in the ConA model, IL-17A produced mainly by Th17, aggravates liver damage (86), but macrophage depletion prevents the development of lesions, at least in part, by interrupting IL-17A signaling. Moreover, it was shown that in the early stage of liver injury, exosome-mediated TLR3 activation in HSCs aggravates the development of liver fibrosis by enhancing IL-17A Tγδ-cell production in CCl_4_ mouse fibrosis model. HSCs express IL-17A cytokine, and this secretion is TLR3-dependent. TLR3 is activated by an unknown ligand from hepatocyte exosomes (**Table 1**) **(** 21).

**Table 1:** IL-17A in acute liver injury.

Model	Cells	Action	Reference
HepG2 cell line		STAT3 phosphorylation enhances IL-6 secretion	Hu et al. (81)
Viral Infection West Nil Fever (WNF) in IL-17A**^-/-^** mice	Cytotoxic LT CD8	Reduced survival of WNF IL-17A**^-/-^** mice IL-17A sensitize antigen-specific effector T cells	Acharya (82)
Susceptible mice with Theiler’s murine encephalomyelitis virus	Cytotoxic LT CD8	Exhaustive IL-17A response promote viral persistence	Hou et al. (83)
α-GalCer-induced acute hepatitis in mice	NKT IL-17A^+^	Protective role, produced IL-17A inhibits the development of hepatitis.	Wondimu et al. (84)
HBsTg mice ConA lesion	LTγδ IL17-A^+^	Protective, IL-23 mediated role, ameliorated liver damage in HBsTg mice	Meng et al. (85)
WT mice ConA lesion	Th17	IL-17A aggravates liver damage	Yan et al. (86)

#### IL-22

Transgenic IL-22^−^ mice are highly susceptible to increased development of acute hepatitis, and their regenerative processes are delayed (87). The same applies to IL-22BP-deficient mice in a model of toxic hepatitis (88). In a model of acute liver injury (induced by LPS/d-galactosamine), treatment with recombinant IL-22 (rIL-22) has a protective effect due to its anti-apoptotic, anti-inflammatory, and antioxidant effects (89). The same was found for ILC3RORγτ**^+^**IL-22^+^ cells (90). IL-22 overexpression significantly increases the expression of the anti-apoptotic genes *Bcl2*, *Bcl-xL* and the STAT3-p53 axis, induces HSCs senescence, and thus protects the liver from injury in ConA, carbon tetrachloride (CCl_4_) or Fas agonist models (91–94). In a mouse model of acute ethanol-induced injury, treatment with rIL-22 improved alcoholic steatosis, tissue damage, and oxidative stress *via* STAT3 activation (95); and rIL-22 inhibited HSCs activation *in vitro (* 96). All this points rather to the protective side of IL-22 during acute liver injury, directed towards tissue regeneration (**Table 2**).

**Table 2:** IL-17A in chronic liver injury.

Model	Cells	Action	Reference
**Mice**			
IL-17AR-deficient mice CCl_4_-induced fibrosis	Th17, neutrophils IL-17A^+^	Neutrophile influx, inflammation, fibrosis reduction in IL-17AR-deficient mice	Tan et al. (15); Meng et al. (14)
CCl4-induced fibrosis in TLR3-deficient mice	LTγδ IL17-A^+^	Enhanced IL-17A production by LTγδ TLR3-mediated	Seo et al. (21)
BDL-induced model of liver fibrosis, cholestatic model	Th17	IL-17A neutralization improved BDL-induced fibrosis	Zhang et al. (97)
MDR-/- mice, cholestatic model	LTγδ IL17-A^+^	Periportal-bridging fibrosis, increased numbers of LTγδ IL17-A^+^	Tedesco et al. (98)
C57BL/6 mice on high fat diet	Th17	Higher frequency of liver Th17 cells compared to normal diet; inhibition of fatty acid oxidation, steatosis exacerbation	Tang et al. (99); Shen et al. (100)
IL-17AR-deficient mice + high fat diet	CD4^+^IL-17A^+^	IL-17A production exacerbated obesity-induced hepatocellular damage.	Harley et al. (101); Giles et al. (102)
Humanized mice on high fat diet	CD4^+^IL17A^+^	Inflammation, NASH progression, liver fibrosis	Her et al. (103)
**Human**			
Chronic HBV^+^ patients, human liver samples, immunohistochemistry staining	neutrophils IL-17A^+^ CD45^+^IL-17A^+^	Neutrophils IL-17A^+^, CD45^+^IL-17^+^ infiltration of human liver tissue, independent of fibrosis stage.	Macek et al. (104)
Chronic HBV^+^ patients, Human liver biopsies, immunohistochemistry staining, Flow Cytometry	CD4^+^IL-17A^+^	Increased IL-17A expression in advanced fibrotic stage, increased CD4^+^IL-17A^+^ infiltration	Fabre et al. (105); Zhang et al. (12); Tan et al. (15); Wang et al. (106); Zhang et al. (107)
NASH patients	IL-17A^+^ cells Tregs	Increased Th17 infiltration, Il-17A association with hepatic steatosis and proinflammatory response in NAFLD	Tang et al. (99); Rau et al. (108)

### IL-17A, IL-22, and Chronic Liver Injury

If the damaging factor is eliminated, the inflammatory process shifts to regeneration, and, in addition to the regenerative signal provided by IL-22, the switch in macrophage phenotype from pro-inflammatory to pro-fibrotic downstream of the inflammatory response has been reported (109). If the damaging factor (metabolic syndrome, alcohol abuse, chronic viral infection, auto-immune disease) persists, the inflammatory process results in fibrosis instead of regeneration. The sustained inflammation leads to perpetual activation of the HSCs, which undergo a myofibroblast phenotypic transformation: they secrete the components of the extracellular matrix (8). Since MMPs are blocked by the overexpression of tissue inhibitor of metalloproteinases (TIMPs), the matrix invades the damaged tissue. The role of IL-17A appears to be crucial in chronic liver inflammation (**Table 3**). The pro-fibrogenic role of IL-17A has also been reported in pulmonary (111) and intestinal fibrosis (112).

**Table 3:** IL-22 in acute liver injury.

Model	Cells	Action	Reference
**Mice**			
TgIL-22**^-^** mice	Th17 IL-22^+^	Susceptible to the enlarged development of acute hepatitis, protective role of IL-22	Zenewicz et al., (87)
IL-22BP-deficient mice Acetaminophen-Induced liver Injury, toxic hepatitis		Susceptible to the enlarged development of acute hepatitis, protective role of IL-22	Kleinschmidt et al., (88)
LPS/d-Gal, rIL-22 treatment		Anti-apoptotic, anti-inflammatory, and antioxidant actions	Xing et al., (89)
CCl_4_ acute liver injury	ILC3RORγτ**^+^**IL-22**^+^** cells	Protective role of ILC3RORγτ**^+^** IL-22^+^ cells	Matsumoto et al., (90)
CCl_4_ acute liver injury, TgIL-22^+^ mice, rIL-22 administration.	Th17, Th22, Th1	Protective role of IL-22 *via* the induction of HSC senescence.	Kong et al., (93); Lu et al., (94)
ConA lesion T-cell mediated model	LTCD3^+^ lymphocytes	protective role of IL-22 *via* STAT3 activation	Radaeva et al., (110); Pan et al., (91)
Ethanol-induced injury	Th17	Improved liver damage, steatosis *via* STAT3 activation	Ki et al., (95)

#### IL-17A

Active liver fibrosis is accompanied by IL-17A^+^ Th17, myeloid-derived suppressor cells (MDSCs) (99), LTγδ, MAIT, ILC3 cells infiltration (113, 114) in particular in CCl_4_ or BDL-inducing liver fibrosis mice models (14, 115). The IL-17AR–deficient mice that underwent CCl_4_-induced fibrosis, showed a reduction in neutrophil influx, pro-inflammatory cytokines, hepatocellular necrosis, inflammation, and fibrosis compared to control (14, 15). This may be due to NLRP3 inflammasome inhibition (116). In turn, HSCs stimulated by IL-17A increase the secretion of IL-6, TGF-β1, collagen production, α-SMA expression, all markers of HSCs activation (15). However, IL-17A rather sensitizes HSCs for TGF-β-signaling by regulating TGF-β2-receptor expression and does not activate them directly (117). The same was seen in the BDL model (97). Cholestatic mice *mdr* ^−/−^ model shows increased infiltration of the liver by LTγδIL-17A^+^ (98).

A pro-fibrotic and pro-inflammatory role of IL-17A was shown in the NASH mouse model (99, 100). The use of IL-17A–deficient mice models has been shown to improve/resist to the development of steatohepatitis, a major risk factor of fibrosis (101, 102). Humanized mice on high fat diet with induced NAFLD develop liver fibrosis that is mediated by CD4^+^IL17A^+^ cells (103). The depletion of CD4^+^ cells in these mice reduced fibrosis and inflammation but not steatosis. Moreover, in human liver, an increase in the number of IL-17^+^ cells, among intrahepatic CD4^+^ cells, was observed during the transition of NAFLD to NASH. Th17/Treg ratio was significantly higher in NASH patients, and the Tregs count, on the contrary, was much lower (108).

In patients with chronic viral hepatitis, neutrophils accounted for most of the IL-17A^+^ cells, especially in the late fibrosis stage, but the frequency of CD45^+^IL-17A^+^ lymphocytes in liver tissue was independent of the stage of fibrosis (F0–F3) (104). However, an increase in IL-17A expression has been shown in the advanced stages of HBV-related liver disease by the immunohistochemistry on fresh biopsies of HBV^+^ patients (12, 15, 105–107) or patients with non-alcoholic steatohepatitis (99).

IL-17A inhibits autophagy and may promote the development of hepatocarcinoma (118). Zhang et al. (2017) demonstrated that the resolution of BDL- or thioacetamide-induced inflammation and fibrosis after IL-17A neutralization is due to a shift of the “suppressive” immune response in the fibrotic liver toward a Th1-type response, *via* restoration of autophagy activity through inhibition of STAT3 signaling (97). In addition, activation of autophagy in Kupffer cells decreases liver fibrosis *via* suppression of IL-1β expression (119).

#### IL-22

The impact of IL-22 on fibrosis development in chronic liver injury is much more ambiguous (**Table 4**). Since IL-22 promotes survival and proliferation of epithelial cells and shows its protective properties during acute injury, its role differs depending on the duration and progression of the disease. IL-22 is unable to inhibit hepatitis B virus replication, and its neutralization in a model of HBV transgenic mice improved liver damage (12). At the same time, study on pulmonary fibrosis showed lungs infiltration by TγδIL-22^+^ lymphocytes with protective anti-fibrotic potential (127). IL-22 injection protects mice against BDL-induced liver fibrosis (14). In CCl_4_-induced liver fibrosis, IL-22 is capable to slow liver fibrosis progression *via* an increase in anti-inflammatory KCs to pro-inflammatory-KCs ratio (120). However, IL-22RA1 knock-out mice develop mild fibrosis in response to CCl_4_ treatment, and IL-22/IL-17 inhibition leads to reduced fibrosis (105).

**Table 4:** IL-22 in chronic liver injury.

Model	Cells	Action	Reference
Mice			
HBV^+^ Tg mice, IL-22 neutralization	LTCD4^+^IL-22	IL-22 neutralization improves liver damage	Zhang et al. (12)
BDL-induced fibrosis		Protective role of IL-22	Meng (14)
CCL4 -induced fibrosis	M1/M2 Kupffer cells	IL-22 can increase the ratio of M2/M1, protective role of IL-22	Su et al. (120)
CCL4 -induced fibrosis	Th17, Th22	IL-22 deleterious effects on liver fibrosis	Fabre et al. (105)
CXCL1/High Fat Diet-induced NASH		IL-22 blocked hepatic oxidative stress, *via* induction of the antioxidant proteins. Inhibited inflammation in NASH	Hwang et al. (121)
NASH model (mice fed methionine choline-deficient diet)	Th17, Th22, Th1	IL-22 is protective in NASH but only in the absence of IL-17A	Rolla et al. (122)
NASH model (high fat diet) ILC3KO mice	ILC3RORγτ**^+^**IL-22**^+^** cells	IL-22 enhances hepatic lipid metabolism, and have anti-apoptosis activity	Hamaguchi et al. (123)
**Human**			
HBV^+^ patients, liver cirrhosis	-IL-22^+^ cells -Th17	Increased IL-22^+^-cell infiltration, correlation with advanced stages, cirrhotic liver	Fabre et al. (105); Zhao et al. (124)
HCV^+^ patients	IL-22BP	IL22-BP aggravates liver fibrosis in HCV infection, protective role of IL-22	Sertorio et al. (125)
HCV^+^ patients	IL-22^+^ cells	Increased IL-22^+^-cell infiltration depending on fibrosis stage	Wu et al. (126)

IL-22 shows protective traits in mice models of NASH pathology, but only in the absence of IL-17A (122). CXCL1, which regulates reactive oxygen species release by neutrophils and stress kinase activation in a mouse model of NASH, can be altered by IL-22, attenuating NASH progression (121). Recent study has demonstrated that IL-22 is capable to increase lipid metabolism in the liver and have anti-apoptosis activity (123).

Acting through STAT3 activation, IL-22 promotes hepatocyte proliferation and survival, increases HSC senescence (93). As a consequence, chronic inflammation and strong IL-22 signaling, constitutive activation of STAT3, upregulation of anti-apoptotic genes, vascular endothelial growth factor (VEGF) expression, all these factors promote and enhance the development of hepatocarcinoma (128).

Immunohistochemistry analysis of human biopsies shows significant IL-22**^+^** cell infiltration in HBV^+^ patients with liver cirrhosis (105) ^(^ 129 ^),^. Moreover, systemic level of IL-22 is predictive of survival in cirrhotic HBV^+^ patients (130). The high expression of IL-22 in HBV^+^ patients has been found to promote fibrosis progression by inducing intrahepatic migration of Th17 cells *via* decreased hepatic expression of CXCL10 and CCL20 (124). Also, the pro-fibrotic function of IL-22 is associated with an enhancement of TGF-β1-signaling in HSCs in a p38 protein kinase-dependent manner (105). Meanwhile, IL-22 was protective in chronic hepatitis C and schistosome infection, the high level of IL-22BP was associated with aggravation of hepatic fibrosis (125). However, another team showed worsening effects of IL-22 in HCV-infected patients, as manifested by increased infiltration of IL-22^+^ cells, colocalized with α-SMA protein of HSCs in the advanced stages of fibrosis (126).

### Interplay Between IL-17A, IL-22, and RA

The differentiation of the main source of IL-17A and IL-22, the Th17 cells, is dependent on RA signaling. The major cells involved in the production of fibrosis, the HSCs, are a major source of vitamin A, a storage form of retinyl esters packed in lipid droplets.

#### RA and HSCs Activation

HSCs activation is directly linked to the release of lipide droplets with vitamin A storage, which they use as an important energy source for their activation, supported by autophagy, which is a major source of free fatty acids and fuels the activation of HSCs (8). LPS flow enhances autophagy activation and deregulates retinoid signaling (131). During the first phase of HSCs activation, the lipid droplets decrease in size and migrate to the newly formed cell expansions. Retinyl esters in the lipid droplets are replaced by triacylglycerol species. In the second phase, the remaining lipid droplets decrease in size and undergo degradation (132). According to the limited number of studies, lysosomal lipase (LIPA) as well as PNPLA3 have retinyl-esterase activity and are involved in the breakdown of retinyl esters during HSCs activation (65, 133).

At the same time, LRAT is the major enzyme for retinyl esters synthesis. LRAT-deficient mice cannot produce retinyl esters. Thus, HSCs of LRAT-deficient mice do not contain retinoid lipid droplets. The absence of retinoid stockage does not enhance liver fibrosis in BDL or CCl_4_ mice models (134). However, it was discovered that exosome-derived long non-coding RNA-H19 (lncRNA-H19) enhanced RA signaling, which was manifested in increased HSCs activation, increased retinol metabolism, and decreased number of lipid droplets in HSCs (134, 135). Moreover, ADH3 is an important link in this activation, because its inhibition leads to disruption of lncRNA-H19/RA signaling and to HSCs’ inactivation. The role of exosomes in HSCs activation is much more ample and perfectly reviewed by Chen (135). Thus, the explicit role of retinoid lipid droplets and active vitamin A metabolites during HSCs activation remains open.

#### RA and Liver Pathology

A decrease in total retinol and an increase in RA were found in the liver of rats treated with CCl_4_ or thioacetamide (136). RA down-regulates fibrosis markers expression in a rat model of alcoholic liver disease, enhancing the abstinence effect (137).

In patients with NASH or NAFLD (or type 2 diabetes), the serum RA concentration is significantly lower than that in healthy subjects (136) (138). Histologically, the expression of RXRα RNA was inversely correlated with the stage of liver steatosis (138). The protective effect of RXRα is likely to be related to the synergy of action with the PPARγ receptor (139–141). The analysis of vitamin A metabolome in human livers with NASH showed disrupted vitamin A homeostasis, potentially contributing to disease progression. Interpretation of retinoid homeostasis on the basis of indirect markers such as retinol concentrations or mRNA data is probably misleading (142).

#### RA and IL-17A Interactions

On the one hand, RA enhances TGF-β1 signaling in T lymphocytes, through increased expression and phosphorylation of the transcription factor SMAD3 (61). On the other hand, RA downregulates TGF-β1/Smad3 signaling, IL-6 and collagen expression in the tissue parenchyma (131, 143). This results in the decrease of HSCs proliferation and fibrogenic gene expression (144). During liver fibrosis, these are key molecules upregulated by IL-17A, thus it can be speculated that disruption of RA signaling will extend the deleterious effects of IL-17A.

RA interacts with different immune populations, which are involved in the development of liver fibrosis. It upregulates RAE1 expression, NK cell-activating ligand expressed on HSC. NK cells proceed to cytotoxicity and thus regulate the number of formed myofibroblasts (110, 145). On the one hand, RA inhibits IL-17A secretion in cultured Tγδ cells stimulated by IL-1β and IL-23 and in infected mice (autoimmunity model), but does not affect INFγ secretion (146). On the other hand, under the influence of RA, Tγδ lymphocytes can secrete large amounts of IL-22, which promotes the affinity of the RAR receptor to the IL-22 promoter, thus reducing inflammation (147, 148). RA also improves liver damage in a T-cell-mediated mouse model by reducing INFγ secretion by NKT cells, but does not affect their activation (149).

RA decreases the expression of IL-6, IL-23, and IRF-4 receptors *in vitro (* 61). This implies that even in the presence of IL-6 and IL-23, there is an increase in FoxP^+^Tregs and inhibition of IL-17A, which can lead to a decrease in neutrophil inflow to the site of inflammation. In *in vivo* EAE models, RA does not increase the frequency of Treg, but was able to inhibit inflammatory response of Th17 cells (61). Lymphocytes T previously treated with RA are no longer able to induce EAE in mice, there is a decrease in the infiltration of the central nervous system by IL-17A**^+^**T cells (146). Moreover, vitamin A supplementation has been shown to decrease RORγτ and IL-17A expression in multiple sclerosis (150).

Another team reports a decrease in liver damage in the cholestatic mouse model (BDL) after treatment of mice with ursodeoxycholic acid (UCDA), the only drug approved for the treatment of liver cholestasis, in combination with RA (151). RA alone or in combination with UCDA significantly reduced the expression of TGF-β1, Col1A1, MMP-2, α-SMA, CYP7A1, TNFα, and IL-1β (151). The same results were seen in the murine model of hepatic fibrosis CCl_4_, administration of RA decreasing TGF-β1 and IL-6 secretion and increasing survival (152).

IL-17A activates the expression of the MMP-2 and MMP-9 *in vitro* (153), which are widely implicated in the progression of liver fibrosis (154). RA, for its part, is able of reversing this activation, thus moderating the spread of the pathological process (fibrosis or cancer) (155). In a rat model of alcoholic liver disease, RA treatment downregulated MMP-2 and MMP-9 expression, as well as TIMPs expression (137), but could enhance MMP-3 and MMP-13 expression in HSC rat cell line (144). In addition RA can promote the upregulation of MMPs in dendritic cells (156) or mesenchymal stem cells (157) causing their enhanced migration to the site of injury.

## Conclusions

There is much evidence of the deleterious effects of IL-17A on the development of a liver disease, particularly liver fibrosis. Regardless of regeneration or fibrosis, the liver responds to damage by activating HSCs that release their vitamin A stores. Active metabolites of vitamin A, such as RA, are strongly involved in the differentiation of the Th17 cell population, the main producers of IL-17A and IL-22. As a result of its anti-inflammatory and immunomodulatory effects, RA can down-regulate the secretion of IL-17A by immune cells and promote IL-22 signaling. However, very few studies have examined the relationship of this IL-17A-AR-IL-22 trio in the context of liver fibrosis.

**Figure 3:**
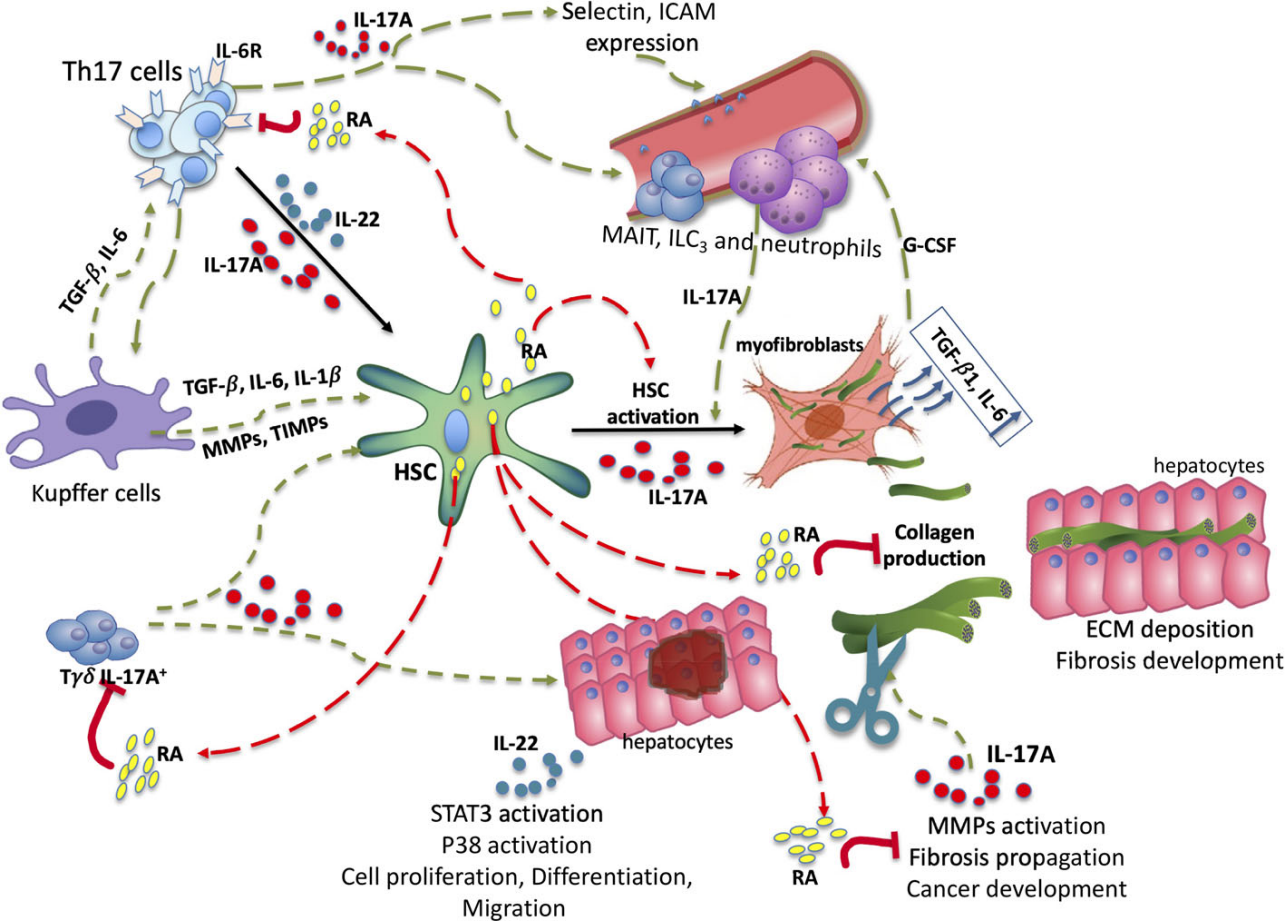
Trio IL-17A/RA/IL-22 in development of liver fibrosis. Under the influence of damaging factors there is an activation of the intrahepatic immune system guided by IL-17A, TGF-β1 and IL-6. Under TGF-β1 and IL6 secreted by Kupffer cells, as well as IL-17A secreted by Tγδ, Th17, myeloid populations, there is an activation of HSC. Upon activation, the HSC releases lipid droplets filled with retinyl esters and transforms into myofibroblasts, generating the extracellular matrix, notably collagen. IL-17A promotes the migration of circulating immune cells. RA is able to disrupt IL-17A, TGF-β1 and IL-6 signaling by inhibiting the expression of IL-6 receptors, thereby moderating HSCs activation. Fibrosis progression is associated with permanent remodeling of the deposited matrix. RA is capable of inhibiting MMP-2, MMP-9, certain TIMPs, and thus moderating the spread of fibrosis. Through STAT3 activation IL-22 contributes to hepatocyte proliferation, differentiation and migration. HSC, hepatic stellate cell; RA, retinoic acid; ECM, extracellular matrix; MMP, metalloproteinase; TIMP, tissue inhibitor of metalloproteinase; ILC3s, type 3 innate lymphoid cells; MAITs, mucosal associated invariant T cells. Red arrows—possible inhibitory effect, Green arrows—possible activator effect.

In addition to a role in the immune component, genetic variations in enzymes, regulating the availability of active Vitamin A metabolites in the liver, have been found. These SNPs affect the prognosis of the course of chronic liver disease, like alcoholic and nonalcoholic liver diseases. In animal models, RA is able to inhibit IL-17A secretion, IL-6R, IL-23R expression, regulate MMPs/TIMPs and TGF-β1 expression, and thus regulate the development of inflammation and fibrosis (**Figure 3**). The data suggest that the concentration of RA in the liver increases progressively as fibrosis progresses and decreases in the serum. This, on the one hand should alleviate IL-17A-associated inflammation, but, on the other hand means a high activation of HSCs and, consequently, advancement of the disease. In addition, according to the data, there is an increased activity of CYP26A1, the enzyme responsible for RA degradation. RA signaling could also be deregulated. There is a need to explore the possible interaction between IL-17A and RA in the liver, to understand whether IL-17A-associated inflammation can be reversed by the action of active metabolites of vitamin A in humans, and to unravel the molecular mechanisms behind this likely regulation. Based on the immunopathobiology of human fibrogenesis, we can speculate that immune modulation of the IL-17A/RA axis could be a new important component of the very active therapeutic development of NASH and fibrotic disease.

## Author Contributions

DK-E, SP, and SL contributed to conception and design of the study. DK-E organized the database, analyzed and wrote the first draft of the manuscript, did the figures, and did the editing. SP and SL contributed to funding acquisition, supervision, and reviewing. All authors contributed to the article and approved the submitted version.

## Funding

This work is supported in part by Assistance Publique-Hôpitaux de Paris (AP-HP, France), by the Institut National de la Santé et de la Recherche Médicale (INSERM, France) and by Institut Pasteur (Paris, France). Daria M. Kartasheva-Ebertz received a PhD Fellowship from Assistance Publique-Hôpitaux de Paris (AP-HP, France).

## Conflict of Interest

The authors declare that the research was conducted in the absence of any commercial or financial relationships that could be construed as a potential conflict of interest.

## Glossary

**Table:**

HCC	hepatocellular carcinoma
NASH	non-alcoholic steatohepatitis
NAFLD	non-alcoholic fatty liver disease
RA	retinoic acid
HSCs	hepatic stellate cells
PRRs	pattern recognition receptors
TLR4	toll-like receptor 4
LPS	lipopolysaccharide
LSEC	liver sinusoidal endothelial cells
TGF-β	transforming growth factor
ECM	extracellular matrix
PD-L1	programmed death-ligand 1
Th17	T helper 17 lymphocytes
Tc17	T cytotoxic 17 lymphocytes
LTs	lymphocytes T
NKT	natural killer T cell
ILC3s	type 3 innate lymphoid cells
MAITs	mucosal associated invariant T cells
GM-CSF	granulocyte colony-stimulating factor
EAE	experimental autoimmune encephalomyelitis
BDL	bile-duct ligation
RADLH	retinaldehydrogenases
RBP4	retinol binding protein 4
RARα, β, γ	retinoic acid receptors
RXRα, β, γ	retinoid X receptors
TNFα	tumor necrosis factor α
IRF-4	interferon regulatory factor 4
AKR1B10	aldo-keto reductase family 1 member B10
ALDH1A	aldehyde dehydrogenase 1 family member A
BCO1	beta-caroten oxygenase 1
SCARB1	scavenger-receptor class B 1
PNPLA3	patatin-like phospholipase domain-containing protein 3
HSD17B13	17β-Hydroxysteroid dehydrogenase type 13
WT	wild type
CCl_4_	carbon tetrachloride
PDGF	platelet derived growth factor
INF -4	interferon 4
G-CSF	granulocyte colony-stimulating factor
ConA	concanavalin A
MMP	metalloproteinase
TIMP	tissue inhibitor of metalloproteinases
MDSCs	myeloid-derived suppressor cells
VEGF	vascular endothelial growth factor
UCDA	ursodeoxycholic acid
